# Notes About Comparing Long-term Care Expenditures Across Countries: Comment on "Financing Long-term Care: Lessons From Japan"

**DOI:** 10.15171/ijhpm.2019.87

**Published:** 2019-10-14

**Authors:** Johannes Geyer

**Affiliations:** DIW Berlin, Berlin, Germany.

**Keywords:** Long-term Care, Social Insurance, International Comparison, Public Expenditures

## Abstract

The comparison of long-term care (LTC) expenditures is a difficult task. National LTC systems differ widely in terms of eligibility criteria, level of benefits, institutional variety and regional heterogeneity. In this commentary I will first give some general remarks on cross country comparisons. Then I discuss the role of the informal sector which is the most important pillar of all LTC systems. I conclude with some background on current developments in Germany. Different from Japan Germany is extending its LTC insurance instead of containing costs.

## Introduction

The editorial “Financing long-term care: lessons from Japan”^1^ by Naoki Ikegami [henceforth NI] raises many important points regarding long-term care (LTC) systems, the role of the government and experiences in Japan. I discuss selected points made by NI and complement his contribution by discussing current issues related to Germany’s LTC system.

## Long-term Care Expenditures

### General Remarks

Comparing LTC expenditures across countries is a difficult task. For example, LTC systems differ in many dimensions and it might not always be possible to unambiguously identify LTC services delivered by health care institutions and social services. Fortunately some data sources for international comparison are available. For example, the Organisation for Economic Co-operation and Development regularly publishes comparable data on LTC expenditures.2 Measuring expenditures as percentage of gross domestic products (GDP) these data show almost the same ranking as reported by NI. Data from 2017 show that Sweden spends about 3.5% of GDP^ [1]^ on LTC followed by Japan^ [2]^ (2.3%), United Kingdom (2.3%), Germany (2.2%), Italy^ [3]^ (1.7%) and the United States (0.9%). Depending on the specific research question these numbers may not be sufficient for a cross-country comparison of expenditures. For example, not all LTC services are devoted to elderly care but may include services for younger age groups as well.^4^ If the goal is a comparison of public resources spent on elderly care one has to take that into account. Another problem for the international comparison is the internal organization of the LTC sector in a given country. For example, Italy’s LTC system differs strongly across regions and is characterized by strong institutional fragmentation.^5,6^

### Composition of Expenditures

NI emphasizes that the “key issue in LTC is not necessarily costs, but resource allocation” (NI, p2). Comparing Germany, Italy and the United Kingdom, he is surprised to find higher expenditures in Italy and the United Kingdom since they do not have formal LTC programs as in Germany. Germany is spending relatively more on formal care services than the other two countries which spent more on cash benefits. However, it depends on the research question whether this information is helpful or not. There is no reason why a formal LTC program has to be more expensive than a system that mainly relies on means-tested safety-net schemes^ [4]^.

### Private Costs and Other Outcomes

I would disagree about the claim that private LTC expenditures are impossible to estimate and, therefore, should not be considered in a country comparison. I think this depends on the research question. In general I would rather emphasize that an international comparison of LTC expenditures should take into account the informal sector. At least it should be discussed. There is a huge literature that shows how different LTC systems are. Depending on the LTC system the informal LTC varies a lot across countries.^7^ If we ignore the informal sector important costs of LTC systems will be hidden and the comparison will be incomplete. For example, LTC services may enable informal caregivers to stay employed instead of withdrawing from the labor market.^8^ As a consequence taxes and social security contributions compensate for some of the LTC expenditures.^9^ And low public expenditures may entail high private costs such as co-payments, reduced employment, lower mental and physical health.^10^ There is also a literature that tries to estimate and quantify the contribution of the informal sector to the economy.^11-15^

It may be difficult to estimate these costs but it is not impossible. In Germany, for example, the cost of housing in residential care are not covered by the LTC insurance. Data on these co-payments are available and could be used to calculate private costs. Moreover, surveys asking questions on LTC have improved and often ask for costs associated with informal care provision^[5]^. Another way to estimate costs on informal care is to estimate labor supply reactions and to calculate forgone earnings, taxes and social security contributions.

In addition to private costs, it would be interesting to complement expenditure data with health outcomes of people in need of LTC and comparable quality measures of LTC facilities. This would help to assess the efficiency of spending in different countries. Again, this is difficult and comparable data are hard to find but it would improve such studies enormously.

### Goals

The article emphasizes that the goal of LTC is to compensate for the decline in functional capacity and to mitigate the care burden of the family. However, LTC is also part of a country’s welfare system and provides a an insurance scheme where private markets usually do not exist or do not function well.^16^ The cost of formal care can be very high for private households. Moreover, there are significant uncertainties about the LTC risk, the severity of LTC needs and its duration.^17^

### Government’s Responsibility

I would like to add two points regarding the German LTC system. First, Germany comprehensively reformed its LTC system in the years 2015-2017. The most important change is a new assessment scheme moving from a focus on physical impairments to a complex scale which measures self-reliance. This step was necessary since the number of people with cognitive impairments (dementia) increased and the previous scheme did not sufficiently account for this group. The new assessment scheme takes into account six areas of daily life:

1. Mobility (10%)
2. Cognitive and communication abilities (higher value from module 2 and 3, in total 15%)
3. Behavior and psychological problems (higher value from module 2 and 3, in total 15%)
4. Self-care (40%)
5. Dealing with requirements due to illness, therapy or medication (20%)
6. Organization of everyday life and social contacts (15%)

The number of beneficiaries increased – mainly due to the reform – between 2016 and 2018 from 2.75 Mio. to 3.8 Mio. Expenditures increased from 31 Bil. Euro in 2016 by 32% and reached 41 Bil. Euro in 2018.

The second point is the difficulty in maintaining a sufficiently large LTC workforce to meet the growing demand for LTC services. This seems to be one of the most important problems in the near future.^18^ Most likely Germany will experience a growth in LTC demand that will be larger than the growth in informal care supply. Currently, the government tries to increase the attractiveness of jobs in the LTC sector. For example, the minimum wage for unskilled worker in the LTC sector was increased in 2019 and will be further increased in 2020. However, such measures will eventually lead to higher expenditures.

### Containing Costs or Developing the LTC Sector

For a long period, we have not seen reforms in Germany aimed at containing costs like in Japan. The current strategy is rather to extend the LTC insurance, to lower private costs and to increase support for family caregivers. For the time being the German LTC system remains a mixture of a universal public insurance scheme and informal care by family members. However, this strategy will be difficult to maintain in the long-run. The expected increase in demand for LTC is a great challenge for the system. Informal caregivers are expected to remain the main care workforce in the future and a much higher share of the working-age population will have to provide care while being employed. The alternative would be to extend the public insurance scheme moving to a full-coverage insurance scheme which would heavily increase public expenditures.

## Ethical issues

Not applicable.

## Competing interests

Author declares that he has no competing interests.

## Author’s contribution

JG is the single author of the paper.

## Endnotes


[1] OECD data show that only the Netherlands spend more on LTC (4%). I included health and social components of LTC expenditures.



[2] Data from 2014.



[3] The OECD data for Italy seem to be incomplete. They report a GDP share of only 0.7% which is much lower than in any other report. The reported 1.7% is taken from the recent Ageing Report by the European Commission and refers to the year 2016.^3^



[4] The cost of LTC systems also depend on whether a country’s economy is labor or capital intensive. Since low- and middle-income countries are generally labor intensive and LTC is a labor-intensive service, the costs of LTC in these countries are relatively lower in comparison to costs in high-income countries, but the formal care sector there is usually underdeveloped.



[5] For example, Health Survey for England asks older people whether they receive privately funded social care, and the German Socio-Economic Panel Study asks for private expenditures associated with LTC if a person in need of care lives in the household. Combined with other individual-level information in the survey such as hours of care, level of activities of daily living per instrumental activities of daily living disability, and type of helpers, research can now get a better estimate regarding the volume of private care.

